# Parkinson Subtypes Progress Differently in Clinical Course and Imaging Pattern

**DOI:** 10.1371/journal.pone.0046813

**Published:** 2012-10-08

**Authors:** Carsten Eggers, David J. Pedrosa, Deniz Kahraman, Franziska Maier, Catharine J. Lewis, Gereon R. Fink, Matthias Schmidt, Lars Timmermann

**Affiliations:** 1 Department of Neurology, University of Cologne, Cologne, Germany; 2 Department of Nuclear Medicine, University of Cologne, Cologne, Germany; 3 Cognitive Neurology Section, Institute of Neuroscience and Medicine (INM-3), Research Centre Jülich, Jülich, Germany; Baylor College of Medicine, Jiao Tong University School of Medicine, United States of America

## Abstract

**Objective:**

To elucidate whether Parkinson’s disease (PD) subtypes show a differential pattern of FP-CIT-SPECT binding during the disease course.

**Methods:**

We examined 27 patients (10 female, 17 male, mean age 61.68±11.24 years, 14 tremordominant, 13 akinetic-rigid) with [^123^I]FP-CIT-SPECT and clinical ratings including UPDRS III after at baseline and after a mean period of 2.47 years. Patients had been classified at baseline as tremordominant or akinetic-rigid according to a “tremor score” and “non-tremor score”. These subgroups were compared for differences in disease progression. Means of clinical ratings and the quantitative analyses of FP-CIT-SPECT for ipsi- and contralateral putamen and caudate nucleus were calculated and compared between baseline and follow-up.

**Results:**

There were no statistical differences concerning age, disease duration, L-Dopa equivalent dose, disease severity (UPDRS III) or dopaminergic uptake in FP-CIT-SPECT at baseline between both subgroups. At follow-up, akinetic-rigid patients showed a distinct and statistically significant reduction of the dopaminergic uptake associated with significant progression of the clinical symptoms (UPDRS III). In contrast, in tremor patients the aggravation of clinical symptoms and dopaminergic deficit was less pronounced without statistical significance among assessments.

**Conclusions:**

This study shows for the first time a considerable progression of clinical symptoms and in-vivo dopaminergic deficit of akinetic-rigid compared to tremordominant PD patients over time. Our data may help to improve strategic planning of further therapeutic trials and to provide a clearer prognosis for patients regarding the perspective of their disease.

## Introduction

The widely used term of idiopathic Parkinson’s disease (PD) comprises motor and non-motor deficits which progress over time. The motor symptoms include bradykinesia, rigidity, tremor and postural instability. Non-motor symptoms such as depression, dementia, autonomic dysfunction or sleep disorders are recognized nowadays as additional important features of the disease. Neuro-pathological changes affecting complex cerebro-basal-ganglia loops have been shown to underlie these motor- and most of the non-motor features of PD. [1], [2].

The phenotype of PD is thus heterogenous and can be classified into different clinical subtypes. Following the most prominent motore features, akinetic-rigid, tremordominant and equivalent subtypes have been defined. [3] Clinical observations suggest that distinct subtypes of PD have a different clinical course. [4], [5], [6] Patients with an akinetic-rigid subtype show a faster clinical progress associated with more severe cognitive decline. [4], [7] These data were confirmed amongst others by Rajput and colleagues [4] in a clinicopathological study which showed that the more favorable outcome of tremordominant patients is related with a less widespread pallidal and striatal reduced dopamine level compared to akinetic-rigid PD patients.

[^123^I]FP-CIT-SPECT (DaTSCAN, Amersham Health, UK) images dopamine transporters. Loss of dopamine transporters shows a good correlation with PD staging, severity, disease duration and the nigrostriatal deficit of patients suffering from PD at post-mortem. [8], [9] Thus, the FP-CIT-SPECT is a widely accepted method to image in-vivo the dopaminergic neurodegeneration in PD. Previous studies of PD subtypes gained inconsistent data regarding the differences in dopaminergic uptake at a given time point. An association between quantitative striatal dopaminergic uptake and PD subtype has not yet been established. [10], [11], [12], [13] In an earlier study we could demonstrate that distinct subgroups carefully matched for age, sex, disease duration and L-Dopa equivalent dose had no significant difference in the quantitative dopaminergic uptake in the FP-CIT-SPECT, but showed a significant association of visually analysed shapes of the striatum in FP-CIT-SPECT and clinical PD subtype. [14] We hypothesized that in the progressing disease the akinetic-rigid patients would show a stronger decline in their motor functions. This clinical progress should be reflected in a more pronounced decline of dopaminergic uptake contralateral to the clinically more affected side in the FP-CIT-SPECT. Therefore, after a mean follow-up period of 2.47 years, we re-examined our disease-severity and -duration matched cohort of PD-patients by a broad assessment of clinical parameters and nigrostriatal function as assessed in FP-CIT-SPECT.

## Methods

### Ethics Statement

The ethical committee of the medical faculty of the University of Cologne approved the study (EK 11-081) and all patients gave their written informed consent before participation. Besides, the study was registered in the DRKS (German Clinical Trials Register; trial number DRKS00003110) according to the WHO trial registration guidelines.

### Participants and Clinical Assessment

In a first retrospective analysis, our group consisted of 46 patients (31 males, 15 females; mean age 69.9±11.1 years), who were divided into two subgroups of tremordominant and akinetic-rigid subtypes. These two subgroups showed no significant statistical difference in age, disease-duration, disease severity (Hoehn & Yahr grade [15], Unified Parkinson’s Disease Rating Scale score [16]), L-Dopa-equivalent-dose (LEDD) and quantitative FP-CIT-SPECT analysis at baseline. Inclusion criteria at baseline were male and female patients aged 40–80 years with the clinical diagnosis of idiopathic Parkinson’s disease according to the UK Brain Bank Criteria [17], german native speaker and eligible for informed consent. Exclusion criteria included diseases with conditions affecting the cognition (e. g. stroke, tumor etc.). Especially patients with dementia (PANDA [18] <14 points) were excluded. Tremordominant and akinetic-rigid patients were defined according to clinical judgement of two experienced movement disorders specialists. The “tremor score” was derived in a manner similar to Lewis et al. [19] from the sum of UPDRS items 20 (“tremor at rest”) and 21 (“action or postural tremor”), divided by 7 (the number of single sub-items). We did not use the original classification scheme of Jankovic et al. [3] as this one focusses on postural instability and gait difficulty (PIGD) instead of akinetic-rigid subtypes.

The “non-tremor score” was calculated from the sum of UPDRS items 18 (“speech”), 19 (“facial expression”), 22 (“rigidity”), 27 (“arising from chair”), 28 (“posture”), 29 (“gait”), 30 (“postural stability”) and 31 (“body bradykinesia and hypokinesia”), divided by 12 (the number of single sub-items). Patients were classified as tremordominant, if the “tremor score” was at least twice the “non-tremor score”. Vice versa, the akinetic-rigid subgroup included all patients with a “non-tremor score” at least twice the “tremor score”. The classification into two groups was maintained for follow-up analysis. The remaining patients, in whom the “tremor” and “non-tremor score” differed by less than factor 2, were classified as equivalent type. For details of the retrospective analysis and clinical data, please see our previous publication. [14].

For the follow-up analysis all 46 patients were invited for clinical re-examination and repeated FP-CIT-SPECT. Of these, 27 patients (14 tremordominant and 13 akinetic-rigid patients) agreed to participate in the follow-up study. All patients were examined by a movement disorders specialist. UPDRS-III was assessed in the OFF-state after withdrawal of medication for at least 12 hours (therapy with selegiline was discontinued at least 18 hours before FP-CIT application to avoid any interaction of its metabolites at the dopamine transporter [20]). Moreover, patients were filmed and analyzed in an ON-phase after application of a standard soluble L-Dopa dose of at least 200 mg (Madopar LT™, Hoffmann-La Roche AG) or 1.5 times their daily morning dose. The UPDRS-score was evaluated by two blinded video-raters (CE, DP). For clinical details see also Table 1. The mean time between baseline and follow-up examinations was 2.47 years.

**Table 1:** General data of examined patients.

Parameter	Group	Mean	Standard deviation	p-value
Time between baseline and follow-up†	TD	2.66	±0.64^a^	0.186
	AR	2.27	±0.63^a^	
Disease years follow-up‡	TD	7.26	±1.64^a^	0.582
	AR	5.35	±1.62^a^	
Age follow-up‡	TD	61.53	±11.64^a^	0.911
	AR	61.85	±11.27^a^	
LEDD baseline‡	TD	306.42	404.64^a^	0.711
	AR	254.17	269.44^a^	
LEDD follow-up‡	TD	397.56	262.70^a^	0.578
	AR	421.63	311.91^a^	
Gender follow-up	TD	5∶9 (male vs. female)		
	AR	5∶8 (male vs. female)		
Patients treated with deep brain stimulation (DBS)	TD	2 (both STN-DBS)		
	AR	1 (STN-DBS)		

† = paired-sampled t-test.

‡ = t-test for unrelated samples;

a = parametric distribution of values;

TD = tremordominant, AR = akinetic-rigid.

Corrected p-value: p<0.01.

### Data Acquisition and Analysis

In order to prevent accumulation of free radioactive iodine in the thyroid gland, all patients received potassium iodide orally 30 min prior to intravenous administration of approximately 185 MBq [^123^I] FP-CIT (DaTSCAN™, GE Healthcare™). SPECT image data acquisition was performed 3 h post injection [21], [22] with a triple-head rotating gamma camera (Picker Prism 3000) using a low-energy, high-resolution parallel-hole collimator. 120 projections were acquired over an arc of 360° in steps at 3° in a 128*128 matrix and with an acquisition time of 50 seconds per step. The unprocessed projection data were checked with a sinogram and sine display on an Odyssey-FX workstation (Phillips Medical Systems) for possible patient motion and artefacts. The digital images were reconstructed by filtered backprojection using a low-pass filter and corrected with the algorithm for attenuation.

For the automated semiquantitative analysis, HERMES BRASS™ was used on a Hermes workstation (Nuclear Diagnostics, Stockholm, Sweden) to analyse the dopaminergic deficit. This is a three-dimensional approach which relates the uptake to a normal image template. BRASS™ automatically fits the patient’s image data to a reference template created from healthy controls. This is followed by placing predefined three dimensional volumes-of-interest (VOI) for the quantification of specific to non-specific binding for striatum, caudate, putamen and occipital cortex. [21], [23] The automated fitting algorithm includes an adjustment of the VOIs to compensate anatomic variation. As manual ROI-based approaches have a lower reproducibility, accuracy and higher inter- and intraobserver variability [21], we preferred the automated semiquantitative BRASS™ instead of a quantitative region-of-interest-based analysis.

For the visual analysis of the differential dopaminergic deficit between the two subgroups, we used SPM8 (The Wellcome Trust Centre for Neuroimaging, London, UK) [24] for spatial normalization of all patients on a template within the Montreal Neurological Institute neuroanatomic space (MNI; http:/www.bic.mni.mcgill.ca). The SPECT-template provided by the SPM software package is a cerebral blood flow template which has an intensity profile that differs from that of FP-CIT-SPECT. For this reason, we created our own template consisting of 12 control patients with FP-CIT scans and essential tremor, according to the technique described elsewhere. [25] All individual FP-CIT-scans were normalized using this new template.

### Statistical Analysis

We calculated the means and standard deviation for age, UPDRS-III-ON- and -OFF-score, LEDD and the results of the semiquantitative BRASS™ analyses for the ipsi- and contralateral putamen and caudate nucleus for each subgroup. To detect significant differences between subgroups, we used the student-t-test for independent samples if a parametric distribution was given. When data was non-parametrically distributed the Wilcoxon-Mann-Whitney-test was applied.

Furthermore, we compared the individual differences of disease duration, UPDRS-III-ON- and -OFF-score and the semiquantitative BRASS™ analyses for the ipsi- and contralateral putamen and caudate nucleus between the first and the second examination. In case of a parametric distribution we calculated the mean differences and compared them using a paired sample t-test. If distribution turned out to be non-parametric, we applied a Wilcoxon-signed-rank-test. All statistical computation was performed in PASW Statistics 18 (SPSS Inc., Chicago, Illinois).

To correct for the Type I error for multiple tests between subgroups or time points we used the Bonferroni correction by dividing the set significance level (p<0.05) by the number of tested items. Each corrected p-value can be found below the according tables.

According to our hypothesis, we expected a stronger decline of the dopaminergic uptake in the akinetic-rigid patients. Based on our previously published data [14], the difference between tremordominant and akinetic-rigid patients was most pronounced in the putamen. For this reason we used an a-priori hypothesis-driven “small volume approach” for the SPM analysis and compared statistically significant differences of caudate and putamen, instead of using a whole brain analysis. [26].

Voxel-wise statistics were computed using the SPM8 software. All scans were smoothed by a Gaussian filter of 8 mm full width half maximum (FWHM). Subsequently, all images were spatially normalized to a standard stereotactic space by affine 12-parameter transformation using the newly generated template (see above). The two-sample t-test function was used to test for significant group differences. To account for the Type I error, multiple tests were Bonferroni-corrected by dividing the set significance level (p<0.05) by the number of tested regions (4 regions: caudate right/left, putamen right/left).

## Results

After a mean follow-up period of 2.47±0.65 years, serial dopamine-receptor transporter imaging (FP-CIT) was performed on 27 patients (mean age 61.68±11.24 years) suffering from PD. 14 patients with tremordominant subtype of PD at baseline and 13 patients with an akinetic-rigid phenotype at baseline were included. At follow-up, 9 patients were tremordominant, 6 patients showed an equivalent subtype and 12 patients were classified as akinetic-rigid. Both groups were matched at baseline for disease duration, age, LEDD and gender. Regarding these matched items there was no significant difference between both groups. The clinical details for both groups are summarized in Table 1. The baseline data of these patients were taken from a previously reported study [14]. Unfortunately we had a drop out rate at follow-up of about 40% due to loss of contact at follow-up, refusal of consent at follow-up or newly diagnosed concomitant diseases. The individual reasons for each patient are shown in the Table S3. However, the two groups (follow-up group and drop-out group) did not differ statistically significant with respect to L-Dopa-equivalence dose, UPDRS-III motor score in the OFF- and ON-state or age.

**Figure 1:**
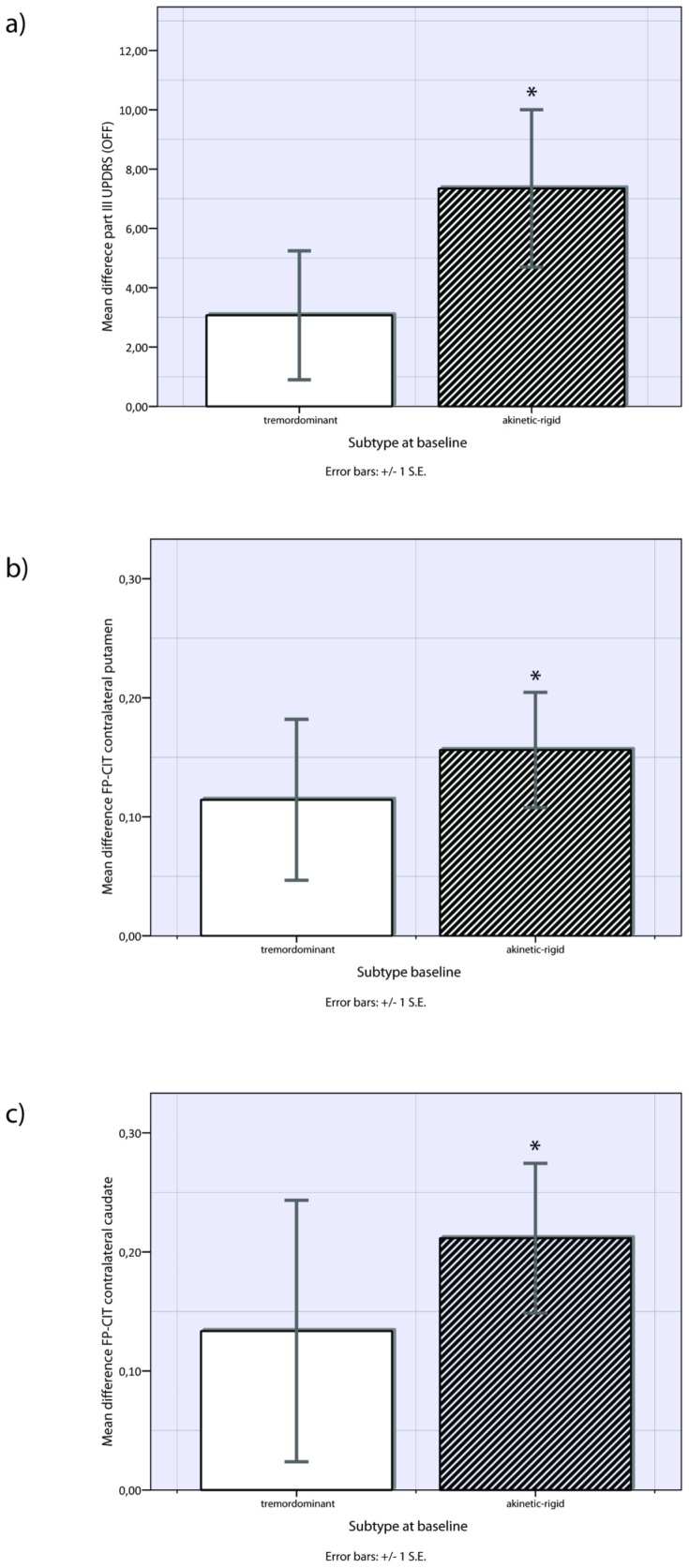
Longitudinal differences in subgroups of PD. a) Mean differences between baseline and follow-up of UPDRS-III-OFF scores for tremordominant and akinetic-rigid PD patients. Significant differences (p<0.05) between the two time points are indicated with *. b) Mean differences between baseline and follow-up of FP-CIT-uptake in the contralateral putamen for tremordominant and akinetic-rigid PD patients c) Mean differences between baseline and follow-up of FP-CIT-uptake in the contralateral caudate for tremordominant and akinetic-rigid PD patients. Significant differences (p<0.05) between the two time points are indicated with *.

At baseline assessment, UPDRS-III motor score did not reveal significant differences both in the OFF- and ON-state between the subtypes (see Table S1). In contrast, the follow-up evaluation showed a remarkably faster clinical progression in the akinetic-rigid compared to tremordominant patients indicating that akinetic-rigid ones were clinically more affected. A paired-sampled t-test showed a significant increase in the mean difference in the UPDRS-OFF-motor score for akinetic-rigid patients (+7.31±7.96; p = 0.012), while tremordominant patients had a smaller increase at the same time, not reaching statistical significance (+3.35±7.85; p = 0.134). Looking at the ON-motor score, similar results were obtained by a Wilcoxon-signed-rank-test: while the akinetic-rigid group increased significantly in motor score (+6.68±8.25;p = 0.023), tremordominant-patients remained stable or even had a lower mean UPDRS-III-ON score without reaching significance (−2.17±9.18; p = 0.391, see Table 2). Mean differences of UPDRS-scores over time are displayed in Figure 1a.

**Table 2:** Differences of the UPDRS III of examined patients over the time course.

Parameter	Group	Mean difference	Standard deviation	p-value
UPDRS-III (ON) baseline vs. UPDRS (ON) follow-up 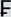	TD	−2.17	±9.18^b^	0.391
	AR	+6.68	±8.25^b^	**0.023**
UPDRS-III (OFF) baseline vs. UPDRS (OFF) follow-up†	TD	+3.35	±7.85^a^	0.134
	AR	+7.31	±7.96^a^	**0.012**

† = paired-sampled t-test,

‡ = t-test for unrelated samples,

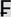
 = Wilcoxon-signed-rank-test;

a = parametric distribution of values;

b = non-parametric distribution of values;

TD = tremordominant, AR = akinetic-rigid.

Corrected p-value: p<0.025.

The analysis of both subgroups with the BRASS™-tool showed a reduced dopaminergic uptake contralateral to the more affected side at baseline without any significant difference neither in putamen nor caudate. The follow-up evaluation still demonstrated no significant difference between both groups (for detailed data see Table S2).

In contrast, for our akinetic-rigid patients we could elucidate a significant decrease in the specific binding of FP-CIT over the course of time, both in the contralateral caudate as well as in the ipsi- and contralateral putamen (mean difference caudate contralateral: 0.22±0.19; p = 0.004; mean difference putamen contralateral: 0.15±0.16; p = 0.019; mean difference putamen ipsilateral: 0.18±0.22; p = 0.011) while tremordominant-patients had no significant results in mean differences of putamen and caudate over time (see Table 3 & Figure 1b&1c).

**Table 3:** Differences of the specific binding of dopamine receptor-transporter (FP-CIT) in striatal regions ipsi- and contralateral to the more affected body side over the time course as examined with the BRASS™-tool.

Parameter	Group	Mean difference	Standard deviation	p-value
Caudate contralateral baseline vs. Caudatecontralateral follow-up 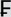	TD	0.11	±0.43^b^	0.272
	AR	0.22	±0.19^b^	**0.004**
Putamen contralateral baseline vs. Putamencontralateral follow-up 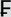	TD	0.10	±0.26^b^	0.184
	AR	0.15	±0.16^b^	**0.019**

† = paired-sampled t-test,

‡ = t-test for unrelated samples,

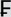
 = Wilcoxon-signed-rank-test;

a = parametric distribution of values;

b = non-parametric distribution of values;

TD = tremordominant, AR = akinetic-rigid.

Corrected p-value: p<0.025.

**Figure 2:**
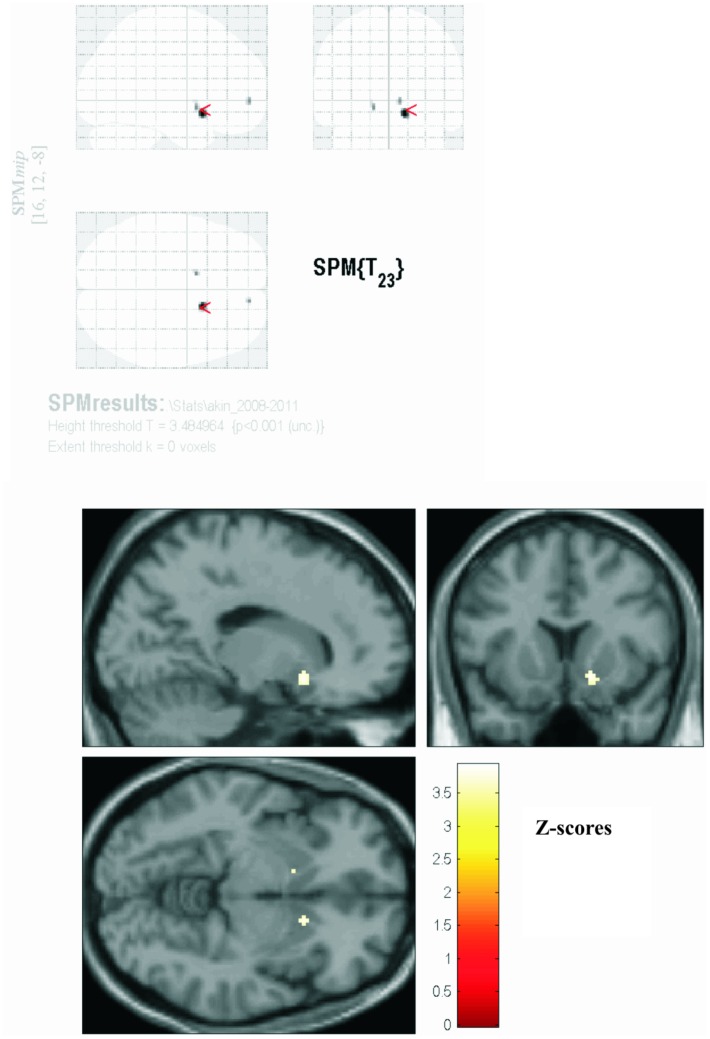
SPM results for akinetic-rigid PD patients. Results of the SPM-analysis superimposed on a standard MRI-scan for akinetic-rigid patients show a significant reduction of the dopaminergic uptake between baseline and follow-up.

SPM statistics revealed a significant cluster of reduced dopaminergic uptake in the right (p<0.001) and left (p = 0.001) putamen for akinetic-rigid patients over time (see Figure 2), whereas tremordominant patients did not show a statistically significant difference in the same period. Between group statistics of akinetic-rigid and tremordominant patients revealed no significant differences at baseline and follow-up.

## Discussion

In this study, age, disease-duration, disease-severity and LEDD-matched groups of akinetic-rigid and tremordominant PD patients did not differ significantly at baseline. In contrast, standardized semiquantitative analysis of FP-CIT-scans differed with regard to the pattern of dopaminergic loss. The visual analysis showed a significant association of tremor-dominant patients with eaglewing-shaped and akinetic-rigid with egg-shaped striatal configurations. [14] After a mean follow-up period of 2.47 years akinetic-rigid patients showed a distinct progression of clinical markers and dopaminergic deficit in FP-CIT-scans. The progression of dopaminergic loss was most explicit in the putamen bilaterally. The data demonstrate, that the predominant clinical PD subtypes are associated with differential dopaminergic degeneration.

The dopaminergic deficit underlying PD as imaged by PET or SPECT has prevailed as a biomarker of the dopaminergic deficit and of disease progression over time. The annual decline of dopaminergic uptake in FP-CIT-scan is about 8%. [27] Overall, imaging and post-mortem studies show a more distinct decline in dopamine depletion in the putamen than in the caudate nucleus, reflecting an anterior-posterior gradient. [28], [29], [30] This gradient of dopamine dysfunction has been shown from early disease stages onwards and does not change substantially during disease progression. [30] To the best of our knowledge, no effort has been made so far to distinguish different PD subtypes regarding imaging progression patterns over time.

In our previous study, we could demonstrate different visual patterns of FP-CIT-uptake in tremordominant and akinetic-rigid PD patients. [14] These findings suggested a different pattern of Dopamine-loss which might reflect different neuropathological features associated with the disease subgroups. The sequential functional imaging in this study permits the demonstration of individual longitudinal progression in the FP-CIT-scan and could show a more pronounced decline of dopaminergic uptake in the akinetic-rigid subgroup. This reduced uptake was most pronounced in the caudate contralateral to the clinically most affected side and the ipsi- and contralateral putamen. Different neuropathological patterns for PD subtypes that may underlie these differential patterns of FP-CIT-uptake over time could be demonstrated. [4], [31] Additionally, Selikhova et al. showed a substantially different cortical involvement in PD subtypes. [32] However, there is no fully established neuropathological model for the dopaminergic progression of subtypes over time. The decay of both putamina in the akinetic-rigid patients might point out the relevance of the putamen in the initiation of a more “malign” course of the disease and could than serve as an indicator for faster progression of the disease. Taken together, the connection of clinical hallmarks, in-vivo imaging data and neuropathological correlates is pending and the lack of a longitudinally assessed, autopsy verified cohort remains a major challenge to be overcome in future studies.

The classical scheme for subtyping of PD patients consists of the subgroups tremordominant, akinetic-rigid and equivalent type. Besides this standard classification, there is a vast diversification of subtype classification schemes. Recently, there have been different attempts to refine these subgroups using empirical approaches such as cluster analysis or latent class analysis. The data-driven techniques search for clusters of patients with low intra-group but high inter-group differences between selected variables and do not predetermine clinically or theoretically defined subgroups. [33].

A metaanalysis of van Rooden and coworkers reviewed the broad clinical spectrum in PD and found, as the main overlap in the majorities of studies, the cluster profiles “old age-at-onset and rapid disease progression” and “young age-at-onset and slow disease progression”. [34] Other studies defined subgroups with young onset, tremordominant, non-tremordominant and rapid disease progression or postural instability/gait difficulty and tremordominancy. [19], [35], [36], [37], [38] Regardless of the method used there is clear evidence that under the “umbrella of Parkinson’s disease” a large clinical heterogeneity with different progression and prognosis over time exists. We retained the subgroups tremordominant and akinetic-rigid since we started the patient classification approx. four years ago, when large cluster analyses for subtypes where not yet established, and we did not want to change post-hoc the classification scheme used at baseline.

These data imply different pathophysiological mechanisms of PD subtypes which are in need of different treatment strategies. PD gradually affects activities of daily living and has a negative impact on health-related quality of life (HRQoL). HRQoL is relatively preserved in tremordominant patients, in particular at the beginning of the disease. [39] Thus, patient management should account for the clinical subtype. For research purposes, these patients may have to be differently stratified for clinical trials, e.g. studies aimed to evaluate disease modifying (e. g. neuroprotective) therapies. Our data strongly support such a notion demonstrating differential dopaminergic loss over time across both subgroups.

There are a couple of limitations to consider in this study. Predetermined assumptions about clinical subgroups may lead to a bias in the conclusions. Data-driven approaches without assumptions about the defining clinical features can minimize this effect. As it was not a main goal of this study to establish a new subtype classification system, we defined the subtypes according to the classical clinical impression of tremordominancy or akinesia/rigidity. This “standard” classification may have the disadvantage to be “blind” for further differential changes within these cluster-subtypes.

We observed an improvement of UPDRS-ON scores in tremordominant patients over time. These findings may be surprising at first glance. The improved scores are a result of a) an optimized medical treatment or b) due to the effects of deep brain stimulation in two patients. As the OFF-scores demonstrate a decline of the UPDRS in both groups, the overall findings are not counterintuitive.

Another point is the availability of only two data points. As was shown in previous studies (e.g. [30]) three and more follow-ups are more appropriate to demonstrate a “curve of progression”. We only had two data points available and could already demonstrate a differential decline of dopaminergic uptake and disease progression between the two PD subgroups. Moreover, this distinct decline of FP-CIT-uptake is – compared to a nearly linear 6–10% decline of the striatal uptake ratio **per decade** [40] a very pronounced finding. In future studies we will address the progression over a longer time-period and additional follow-ups.

Even in view of these putative short-comings, the assessment of longitudinal data over a period of 2.47 years in clearly defined subgroups of PD has not been achieved before. Thus, these data are unique, as they demonstrate the progression of dopaminergic loss in distinct subtypes of PD. Since we used an automated image analysis (BRASS™ tool) and therewith avoided the confounding factor of subjective regions of interests, this rater-independent technique has high reliability and delivers robust results. [21], [23].

### Conclusion

This study shows for the first time a considerable clinical and in-vivo progression of akinetic-rigid patients over time, whereas tremordominant patients have a relatively stable course. These data may cast new light on the two different entities of one disease. The additional information of the imaging data might help to improve strategic planning of further therapeutic studies and helps to provide a clearer prognosis regarding the future perspective of the individual disease.

## Supporting Information

Table S1 **Clinical data of examined patients.**
(DOC)

Table S2 **Results of the specific binding of dopamine receptor-transporter (FP-CIT) in striatal regions contralateral to the more affected body side as examined with the BRASS™-tool.**
(DOC)

Table S3 **Dropped out patients with clinical details and reasons for dropout.**
(DOC)
