# Supplementary material for: Parkinson Subtypes Progress Differently in Clinical Course and Imaging Pattern
Source: PLoS One. 2012 Oct 8;7(10):e46813. doi: 10.1371/journal.pone.0046813 (PMC3466171; doi:10.1371/journal.pone.0046813)
Supplement: Table S1 — Clinical data of examined patients. (DOC) [file pone.0046813.s001.doc]

Supplementary Table 1: Clinical data of examined patients

| Parameter | Group | Mean | Standard deviation | p-value |
| --- | --- | --- | --- | --- |
| UPDRS-III baseline (ON) ^‡^ | TD | 14.42 | ± 9.02 ^a^ | 0.713 |
|  | AR | 15.86 | ± 9.90 ^a^ |  |
| UPDRS-III baseline (OFF) ^‡^ | TD | 22.64 | ± 11.81 ^a^ | 0.233 |
|  | AR | 29.40 | ± 14.92 ^a^ |  |
| UPDRS-III follow-up (ON) ^‡^ | TD | 13.50 | ± 7.98 ^a^ | **0.013** |
|  | AR | 22.77 | ± 9.26 ^a^ |  |
| UPDRS-III follow-up (OFF) ^‡^ | TD | 26.00 | ± 11.42 ^a^ | 0.051 |
|  | AR | 35.50 | ± 13.58 ^a^ |  |

^†^ = paired-sampled t-test,  ^‡^ = t-test for unrelated samples, ^a^ = parametric distribution of values; TD = tremordominant, AR = akinetic-rigid

Corrected p-value: p < 0.025
