# Supplementary material for: Parkinson Subtypes Progress Differently in Clinical Course and Imaging Pattern
Source: PLoS One. 2012 Oct 8;7(10):e46813. doi: 10.1371/journal.pone.0046813 (PMC3466171; doi:10.1371/journal.pone.0046813)
Supplement: Table S2 — Results of the specific binding of dopamine receptor-transporter (FP-CIT) in striatal regions contralateral to the more affected body side as examined with the BRASS™-tool. (DOC) [file pone.0046813.s002.doc]

Supplementary Table 2: Results of the specific binding of dopamine receptor-transporter (FP-CIT) in striatal regions contralateral to the more affected body side as examined with the BRASS™-tool

| Parameter | Group | Mean | Standard deviation | p-value |
| --- | --- | --- | --- | --- |
| Caudate  contralateral baseline* | TD | 1.36 | ± 0.53 ^b^ | 0.756 |
|  | AR | 1.29 | ± 0.47 ^b^ |  |
| Putamen contralateral baseline* | TD | 0.93 | ± 0.54 ^b^ | 0.458 |
|  | AR | 0.82 | ± 0.60 ^b^ |  |
| Caudate  contralateral follow-up^‡^ | TD | 1.25 | ± 0.54 ^a^ | 0.420 |
|  | AR | 1.08 | ± 0.52 ^a^ |  |
| Putamen contralateral follow-up* | TD | 0.82 | ±0.57 ^b^ | 0.325 |
|  | AR | 0.69 | ±0.51 ^b^ |  |

^†^ = paired-sampled t-test,  ^‡^ = t-test for unrelated samples, * = Wilcoxon-Mann-Whitney-Test; ^a^ = parametric distribution of values; ^b^ = non-parametric distribution of values; TD = tremordominant, AR = akinetic-rigid

Corrected p-value: p < 0.025
