# Supplementary material for: Parkinson Subtypes Progress Differently in Clinical Course and Imaging Pattern
Source: PLoS One. 2012 Oct 8;7(10):e46813. doi: 10.1371/journal.pone.0046813 (PMC3466171; doi:10.1371/journal.pone.0046813)
Supplement: Table S3 — Dropped out patients with clinical details and reasons for dropout. (DOC) [file pone.0046813.s003.doc]

| **Patient number** | **Sex** | **Subtype** | **Disease duration** | **Age** | **UPDRS III OFF** | **Hoehn & Yahr** | **LEDD** | **Reason for dropout** |
| --- | --- | --- | --- | --- | --- | --- | --- | --- |
| 2 | m | 2 | 8 | 69 | 43 | 3 | 1200 | Patient moved to nursery home due to dementia |
| 5 | m | 1 | 7 | 78 | 30 | 2 | 600 | Patient refused participation in the follow-up study |
| 8 | M | 1 | 4 | 73 | 19 | 2 | 500 | Wrong adress, no further contact details |
| 10 | m | 1 | 4 | 68 | 23 | 2,5 | 450 | Patient suffered of colon cancer and was in chemotherapy |
| 11 | m | 2 | 3 | 72 | 27 | 2,5 | 450 | Patient died of cancer |
| 15 | m | 2 | 7 | 62 | 33 | 2 | 600 | Patient refused participation in the follow-up study |
| 18 | m | 1 | 9 | 42 | 46 | 2 | 800 | Wrong adress, no further contact details |
| 20 | m | 1 | 2 | 67 | 12 | 1 | 0 | Patient suffered of paranoid psychosis at follow-up |
| 22 | m | 1 | 5 | 71 | 22 | 2 | 1300 | Development of dementia |
| 23 | m | 1 | 2 | 61 | 15 | 1 | 200 | Patient developed lung cancer; worsening of overall medical condition |
| 25 | m | 2 | 4 | 72 | 22 | 2 | 300 | Patient died of unknown reason |
| 27 | m | 2 | 4 | 70 | 8 | 1 | 300 | Patient refused participation in the follow-up study |
| 32 | m | 2 | 3 | 47 | 33 | 2 | 800 | Wrong adress, no further contact details |
| 33 | f | 2 | 10 | 67 | 41 | 3 | 900 | Wrong adress, no further contact details |
| 36 | m | 2 | 5 | 72 | 28 | 2,5 | 300 | Several lumbar disc prolaps; SPECT examination not possible |
| 38 | m | 2 | 6 | 54 | 18 | 2 | 50 | Development of dementia |
| 43 | f | 1 | 14 | 72 | 49 | 3 | 650 | Patient develooped psychosis after deep brain stimulation |
| 46 | m | 1 | 3 | 52 | 14 | 2 | 300 | Wrong adress, no further contact details |
| 49 | m | 2 | 7 | 63 | 37 | 2 | 50 | Wrong adress, no further contact details |

**Supplementary Table 3:** Dropped out patients with clinical details and reasons for dropout
